# Tumor-associated macrophages: Prognostic and therapeutic targets for cancer in humans and dogs

**DOI:** 10.3389/fimmu.2023.1176807

**Published:** 2023-04-05

**Authors:** Rachel V. Brady, Douglas H. Thamm

**Affiliations:** ^1^Cell and Molecular Biology Graduate Program, Colorado State University, Fort Collins, CO, United States; ^2^Flint Animal Cancer Center, Colorado State University, Fort Collins, CO, United States

**Keywords:** dog (canine), oncology, cancer, immunotherapy, tumor-associated macrophage, tumor microenvironment

## Abstract

Macrophages are ancient, phagocytic immune cells thought to have their origins 500 million years ago in metazoan phylogeny. The understanding of macrophages has evolved to encompass their foundational roles in development, homeostasis, tissue repair, inflammation, and immunity. Notably, macrophages display high plasticity in response to environmental cues, capable of a strikingly wide variety of dynamic gene signatures and phenotypes. Macrophages are also involved in many pathological states including neural disease, asthma, liver disease, heart disease, cancer, and others. In cancer, most tumor-associated immune cells are macrophages, coined tumor-associated macrophages (TAMs). While some TAMs can display anti-tumor properties such as phagocytizing tumor cells and orchestrating an immune response, most macrophages in the tumor microenvironment are immunosuppressive and pro-tumorigenic. Macrophages have been implicated in all stages of cancer. Therefore, interest in manipulating macrophages as a therapeutic strategy against cancer developed as early as the 1970s. Companion dogs are a strong comparative immuno-oncology model for people due to documented similarities in the immune system and spontaneous cancers between the species. Data from clinical trials in humans and dogs can be leveraged to further scientific advancements that benefit both species. This review aims to provide a summary of the current state of knowledge on macrophages in general, and an in-depth review of macrophages as a therapeutic strategy against cancer in humans and companion dogs.

## Introduction

Macrophages are ancient, phagocytic immune cells thought to have their origins 500 million years ago in metazoan phylogeny (1). Ilya Metchnikoff, a Russian zoologist, biologist, and Nobel laureate, first described macrophages in 1882 when he observed large, mobile cells that moved to areas of injury in starfish larvae and phagocytized foreign debris (2). The understanding of macrophages has evolved since then to encompass their foundational roles in development, homeostasis, tissue repair, inflammation, and immunity. Notably, macrophages display high plasticity in response to environmental cues, capable of a strikingly wide variety of dynamic gene signatures and phenotypes. In addition to macrophages derived from blood monocytes, most tissues in the body have resident macrophages uniquely adapted to their local environment.

Regardless of their remarkable diversity, a few core genetic programs establish macrophages’ key role as phagocytes (3, 4). Like other myeloid cells of the innate immune system, macrophages display trained immunity, in which responsiveness to secondary stimulation is enhanced over their first response, and immunological tolerance, in which responsiveness to repeated exposures is diminished (5). Macrophages are also involved in a variety of pathological states including neural disease, asthma, liver disease, heart disease, cancer, and others (6).

In cancer, most tumor-associated immune cells are macrophages, coined tumor-associated macrophages (TAMs) (7). While some TAMs can display anti-tumor properties such as phagocytizing tumor cells and orchestrating an immune response, most macrophages in the tumor microenvironment are pro-tumorigenic (8). Macrophages have been implicated in all stages of cancer. They contribute to the initiation of cancer through smoldering inflammation, leading to a mutagenic environment (9, 10). They support the progression of cancer by inducing angiogenesis, supporting the migration and invasion of cancer cells, and enhancing anti-tumor immunity. They also drive metastasis by preparing the metastatic niche and enhancing tumor cell extravasation and survival during the metastatic cascade (7, 9). Therefore, interest in manipulating macrophages as a therapeutic strategy against cancer developed as early as the 1970s (11–13). Companion dogs are a strong comparative immuno-oncology model for people due to documented similarities of the immune system and spontaneous cancers between the species (14). Data from clinical trials in humans and dogs can be leveraged to further scientific advancements that benefit both species. This review therefore aims to provide a summary of the current state of knowledge on macrophages in general, and an in-depth review of macrophages as a therapeutic strategy against cancer in people and companion dogs.

## Macrophage ontogeny

The understanding of the ontogeny of macrophages has undergone a somewhat dramatic evolution since their initial discovery. Although Metchnikoff popularized macrophages and the theory of phagocytosis, there were earlier observations of phagocytic cells (2, 15). As scientists fought to understand the complexities of the immune system, macrophages were included in an evolving number of classification schemes. The reticuloendothelial system was proposed in 1924, representing a group of cells found in blood vessels capable of forming networks in tissues. Macrophages were observed to differentiate from monocytes in 1925 and documented to respond to sites of inflammation in several experiments in the following decades (16–18). The mononuclear phagocyte system was therefore coined in 1969 as an updated classification scheme, comprised of monocytes and monocyte-derived macrophages (15, 19, 20). Dendritic cells (DCs) would shortly be recognized as distinct cells in the mononuclear phagocyte system as well (21). A bone marrow-derived progenitor cell of monocytes, macrophages and DCs was isolated in 2006 in the mouse, although this finding has since been debated in favor of the theory that there is not a common macrophage-dendritic cell precursor (22, 23). The current understanding indicates that hematopoietic stem cells (HSCs) differentiate into myeloid progenitors and granulocyte-macrophage precursors in adults. A series of intermediates including a common monocyte-restricted progenitor result in mature blood monocytes, which can differentiate into macrophages or monocyte-derived DCs. Other DC subsets likely differentiate from a very early branch point off early myeloid progenitors (24, 25). There is ongoing debate about the exact branching pattern from early progenitors that lead to all known subsets of monocyte, macrophages and DCs (24, 26). It should be noted that monocytes themselves have been recognized as increasingly heterogeneous in origin and function, with multiple defined subsets (26, 27). To help with standardization in the face of this complexity, ontogeny-based classifications schemes of myeloid cells should take priority over schemes that rely primarily on function, location, or cell-surface markers (28). A more comprehensive outline of the history of macrophage research is presented in several good reviews (15, 29).

**Figure 1:**
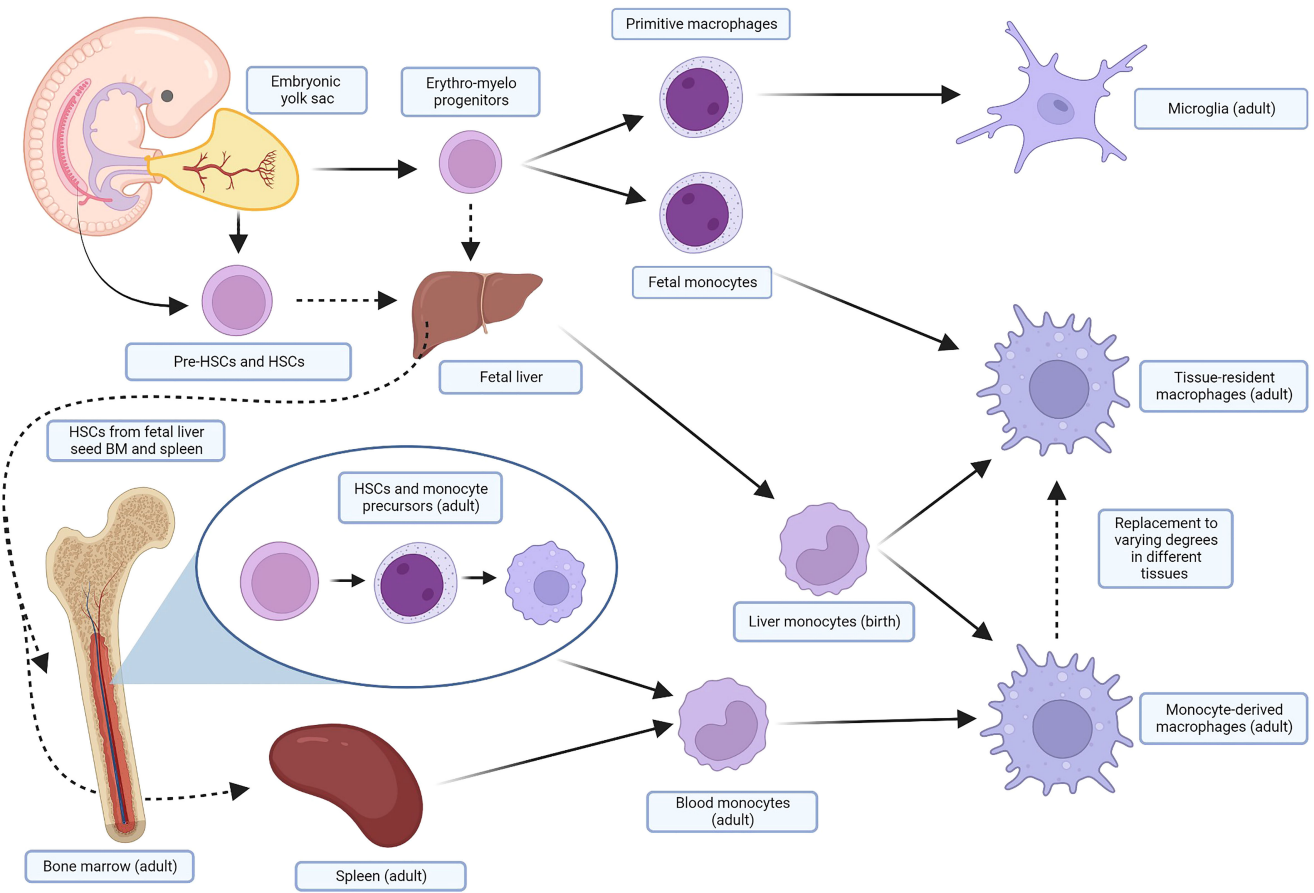
Simplified ontogeny of macrophages. In several waves of primitive hematopoiesis stemming initially from the embryonic yolk sac, erythro-myeloid progenitors differentiate into primitive macrophages and fetal monocytes that eventually become adult microglia and TRMs, respectively. These progenitors also seed the fetal liver, along with pre-HSCs and HSCs from the embryo, yolk sac, and placenta. The fetal liver, the major site of hematopoiesis, subsequently produces liver monocytes, which likely contribute perinatally to macrophage pools in the adult. HSCs, after maturing in the fetal liver, also seed the fetal bone marrow and spleen. In the adult, HSCs from the bone marrow differentiate through several monocyte precursors before being released into the blood as monocytes. These cells are recruited by inflammation and injury in the tissue, and terminally differentiate into MDMS. The spleen also holds a pool of monocytes that can be mobilized when needed. TRMs will be replaced to varying degrees in different tissues by MDMs in the adult. Many of these steps are areas of ongoing research and debate in murine and human models; similar fate-mapping studies have not been undertaken in dogs. For more detail, focused reviews are recommended (34, 40–42). Created with BioRender.com. TRMs, tissue-resident macrophages; HSCs, hematopoietic stem cells; MDMs, monocyte-derived macrophages.

Monocyte-derived macrophages (MDMs) are continually renewed *via* the bone marrow and spleen during periods of high demand such as inflammation or tissue reparation (28, 30). While MDMs remain an important subset of macrophages in vertebrates, in the past two decades a finer understanding of macrophage ontogeny and heterogeneity has evolved. It is now known that the MDMs recruited to inflammation are distinct from tissue-resident macrophages (TRMs). There are numerous populations of TRMs that are singularly adapted to their anatomical location and unique in their ability to self-renew through proliferation (31, 32). TRMs were hypothesized as early as 1971 when Ken Hashimoto identified a resident population of Langerhans cells in the dermis and noted they were self-perpetuating phagocytes (33). Examples of TRMs include the Kupffer cells of the liver, alveolar macrophages of the lung, and microglia of the brain in the adult. Their origins can be traced back to embryogenesis, during which mammalian embryos produce waves of erythro-myeloid progenitor cells prior to the definitive establishment of HSCs in the bone marrow. The yolk sac of the embryo first produces primitive macrophages that develop into microglia in the adult. Subsequent waves of cells from the yolk sac and fetal liver seed resident macrophages in most tissues (34). Fate-mapping studies have shown that these TRMs are replaced to varying degrees by MDMs in the adult (35). Some tissues, like the intestines and skin, appear to rely primarily on MDMs. Other tissues, like the lung, brain, liver, spleen, and peritoneum, rely primarily on their respective TRMs with minimal contributions from MDMs. These balances can be upset after pathological insults (36–38). The exact origin and function of fetal macrophage progenitors is the topic of ongoing debate and is nuanced beyond the scope of this review; several excellent reviews of macrophage ontogeny and nomenclature are recommended (34, 39). A simplified outline of the ontogeny of macrophages is shown in **Figure 1**. The interplay and contribution of MDMs and TRMs to states of health and disease is not yet fully elucidated. Ontogeny, and not just external stimuli, has been established as an important contributing factor to how macrophages respond to physiologic and pathologic stimuli, underlying the importance of further understanding in this area (43, 44).

## Macrophage polarization

Macrophage activation or polarization is a complex topic, in part because of the lack of standardized nomenclature. Both terms are used frequently in macrophage research, without clear definitions for either. Additionally, these terms do not encompass a single switch in a macrophage. Rather, polarization towards a certain phenotype involves multiple steps that trigger changes in concert with an evolving microenvironment (45). In general, activation or polarization refers to the effect of cytokines or growth factors on macrophages, producing changes to their phenotype, metabolism, gene expression, cell surface markers, and function. Macrophage phenotype is determined by a complex network of extrinsic factors, the tissue microenvironment, and genetic and epigenetic regulation (29, 43, 46). Examples of stimuli that impact polarization in addition to cytokines and growth factors include hypoxia, toll-like receptor (TLR) binding, microbes and microbial products, glucocorticoids, tissue damage and nucleotides (29). It is worthwhile to note that many polarization experiments have been performed only in mice and have not been fully replicated in humans (47). A thorough review of the important differences between murine and human macrophages is recommended (48).

The term “activation” was in use by the early 1970s. Peter Alexander and Robert Evans did significant early work with macrophages at the Institute of Cancer Research in London, demonstrating that macrophages could be “armed” and made cytotoxic by exposure to specially sensitized lymphoid cells (13, 49). Veterinarian Isaiah Fidler injected activated macrophages intravenously to inhibit pulmonary metastasis in a mouse model of melanoma around the same time (11). The discovery of specific factors that could activate macrophages was made in the 1980s when it was demonstrated that cytokines such as interferon-gamma (IFN-γ) could increase the antimicrobial activity and oxidative metabolism of macrophages (50). It was thus generally believed that type 1 T helper (Th1) lymphocytes produced IFN-γ to activate macrophages and type 2 T helper (Th2) lymphocyte responses characterized by interleukin (IL)-4 and IL-10 inhibited macrophages. Then, in 1992, Stein et al. described the activation of macrophages by IL-4. As this contrasted with the classical IFN-γ activation, the term “alternatively activated” was used to denote a distinct function and phenotype (51). In 2000, the M1/M2 terminology was formalized in an experiment with Th1 mouse strains (C57BL/6) and Th2 mouse strains (BALB/c) that presented the idea that persists to this day that M2 macrophages have increased arginine metabolism. It was noted that IFN-γ and lipopolysaccharide (LPS) could activate macrophages in the Th1 strains to produce nitrous oxide more easily, while LPS activated arginine macrophage metabolism in the Th2 strains (52). However, a discovery since then has shown that C57BL/6 mice have a mutation that impacts their arginine handling, and therefore the differences seen in arginine metabolism is likely multifactorial (53). Regardless, metabolic reprogramming contributes significantly to macrophage polarization, with differences documented in multiple pathways including glycolysis, arginine metabolism, oxidative phosphorylation, the Krebs cycle, and others. Several good reviews are recommended for more detail (54–56). In 2002 Montovani et al. formalized the knowledge that M1/M2 phenotypes represented “extremes of a continuum of functional states” (57). Presently, the work of transcriptome and single-cell analysis has significantly increased the granularity and depth of the understanding of macrophage phenotype (discussed further below). **Table 1** provides an overview of generally accepted characteristics of the two extremes of macrophage polarization, although as already noted this framework should be applied with caution to *in vivo* systems.

**Table 1:** Two extremes of a spectrum of human macrophage phenotype.

	M1/Classical	M2/Alternative
**Prototypical markers**	CD80, CD86, MHC II, CD64 (54, 58, 59)	CD163, CD204, CD206, FIZZ1 (54, 58, 59)
**Stimuli**	IFN-γ, LPS, TNF-α, TLR ligands (46, 58, 59)	IL-4, IL-10, IL-13, TGF-β (46, 58, 59)
**Secretory products**	TNF-α, IL-1β, IL-6, IL-12, IL-23, CXCL9, CXCL10 (54, 59)	IL-10, TGF-β, CCL17, CCL18, CCL22, CCL24 (54, 59)
**Arginine metabolism**	Metabolizes arginine by iNOS to NO and L-citrulline (54, 56)	Hydrolyzes arginine by arginase to orthenine and urea (54, 56)
**Transcription factors**	STAT1, IRF5, NF-κB (46, 59)	STAT6, IRF4, PPAR (46, 59)
**Function**	Infectious/Th1 response, type 1 inflammation, intracellular pathogen killing, tumor resistance (46, 54, 60)	Allergy/Th2 response, parasite killing, immunoregulation, matrix deposition, tissue repair, tumor promotion (46, 54, 60)

CD, cluster of differentiation; MHC II, major histocompatibility complex class II; FIZZ1, found in inflammatory zone-1; IFN-γ, interferon-gamma; LPS, lipopolysaccharide; TNF-α, tumor necrosis factor-alpha; TLR toll-like receptor; IL, interleukin; TGF-β, transforming growth factor-beta; CXCL, C-X-C motif chemokine ligand; CCL C-C motif chemokine ligand; iNOS, inducible nitric oxide synthase; NO, nitric oxide; STAT, signal transducers and activators of transcription; IRF, interferon regulatory factors, NF-κB, nuclear factor kappa B; PPAR, peroxisome proliferator-activated receptor; Th1, T helper type 1; Th2, T helper type 2.

It is easy to see that these dichotomous labels fall short of capturing the complexity of macrophage reality, which is best described as a multidimensional spectrum (61). One barrier in macrophage research is how to apply results from *in vitro* conditions to *in vivo* reality, as *in vitro* macrophages that are perturbed in a variety of ways merely represent one snapshot in time of these dynamic systems. Experimentally stimulated macrophages *in vitro* have significantly different gene signatures than *in vivo* activated macrophages (62). For example, while granulocyte macrophage colony-stimulating factor (GM-CSF) and macrophage colony-stimulating factor (M-CSF) are often associated with the M1 and M2 phenotype respectively, these growth factors would not be found alone *in vivo* (60). One solution to better capture the complexities of macrophages *in vivo* is to describe additional categories of alternative activation including M2a through 2d (54, 63–66). Other groups have investigated mesenchymal stem cell-activated macrophages as a distinct phenotype as well (67, 68). While the general behavior of these subtypes is largely consistent across experiments, it is easy to find discrepancies in the literature regarding stimuli, markers and secretory products attributed to each (69, 70). As these categories grow, so do the problems of standardization and replication across experiments. In 2014 a group of macrophage researchers informally met to propose unofficial guidelines to attempt to improve clarity and reproducibility within the field (71). They recommend several steps worth summarizing. For example, M1/M2 should only refer to *in vitro* experimental conditions, specifically using IFN-γ and IL-4, respectively, as these activators have been extensively studied (6). They provide comprehensive guidelines on minimum reporting standards for *in vitro* macrophage experiments, including stringent descriptors of the population under study, the isolation and culture method used, and the details of external stimuli used. They also recommend nomenclature that specifies activators, such as M(LPS) rather than M1, to avoid confusion across experiments (71).

Macrophage phenotype has been investigated in dogs. Heinrich et al. used canine MDMs, defining their populations as M0 (unstimulated), M1 (GM-CSF, LPS, IFN γ-stimulated) and M2 (M-CSF, IL-4-stimulated). Using immunofluorescence (IF), only cluster of differentiation (CD)206 was able to distinguish M2-polarized macrophages from the other phenotypes. Routinely used murine and human M1-markers (CD16, CD32, major histocompatibility complex [MHC] class II and inducible nitric oxide synthase [iNOS]) and additional M2-markers (CD163 and arginase-1) were not useful in discriminating the subtypes. Global microarray analysis showed significant differences in the transcriptomes of the polarized macrophage subsets. Interestingly, the identification of gene sets from prototypical literature-based human and murine macrophage gene sets only had minor overlap with gene sets of the polarized macrophages (72).

Herrmann et al. used canine and human MDMs activated with combinations of M-CSF, IL-4, and IL-13 to activate an M2a allergen subtype and GM-CSF, LPS, and IFN-γ for an M1 subtype. On flow cytometric surface marker analysis, only the high-affinity immunoglobulin (Ig) E receptor FcϵR1 was significantly upregulated in canine M2a macrophages, whereas CD86 was upregulated in human M1 macrophages. They did not find significant differences in the expression of another well-established M1-marker CD80 or the M2-marker CD206 in canine MDMs (although CD206 had a statistically insignificant increase in both human and canine M2a macrophages). Reverse transcription-quantitative polymerase chain reaction did show expected changes such as the upregulation of pro-inflammatory genes in the M1 subtype (73).

Chow et al. also investigated canine macrophage phenotype. They cultured canine MDMs in M-CSF and polarized them with IFN-γ (M1) or IL-13 and IL-4 (M2). Flow cytometric analysis of cell surface markers CD86, MHC class II and CD40 were not able to discriminate between M1 and M2 macrophages. Activated macrophages (both M1 and M2) upregulated MHC class II, a finding supported by Heinrich et al. as well (72, 74). Using IF, however, M1 macrophages were shown to significantly upregulate intracellular iNOS, while M2 macrophages upregulated intracellular CD206, transglutaminase 2 and suppressor of cytokine signaling 1 (SOCS1) as compared to M1 macrophages. This group also found significant differences in function as measured by cytokine secretion and phagocytosis, with M2 macrophages displaying an impaired ability to phagocytose and kill intracellular pathogens. (It is worthwhile to note that although it is widely accepted that M2 macrophages have decreased ability to respond to infectious insults, some conflicting data in the human literature describe M2 macrophages as highly phagocytic (75, 76)). RNA-sequencing (RNA-seq) was used to identify unique canine gene signatures of polarized macrophages, resulting in 6 distinct clusters of macrophage genes (74). Macrophage phenotype has also been studied in a variety of canine pathologies, including (non-exhaustively) intestinal disease, spinal cord disease and *Leishmania* infections (77–79).

## Tumor-associated macrophages

Tumors maintain a tumor microenvironment (TME) *via* secretion of cellular factors such as chemokines and cytokines, other environmental factors such as hypoxia and lactic acid, and by the recruitment of local and distant immune and stromal cells. Although most intuitive with solid tumors, blood-borne cancers also maintain a TME composed of a diversity of cells (80). Macrophages are the most abundant immune cell in the TME and comprise a strikingly heterogenous group with multiple ontogenies, phenotypes and roles represented (81). There are differing accounts of the ontogeny of TAMs; both MDMs and TRMs are likely recruited by tumors to varying degrees (82). Some evidence from mice also suggests that a subset of myeloid-derived suppressor cells (MDSCs), a distinct, immature myeloid cell that contributes to the immunosuppression, also differentiate into mature TAMs once in the TME (83–85).There is evidence that subsets of TAMs with differing ontogenies take up different functions in the TME. For example, in a mouse model of lung carcinoma, TRMs were associated with tumor cell growth, while recruited MDMs were more associated with metastasis (86). Even in protected spaces such as the central nervous system, both MDMs and tissue-resident microglia with distinct transcriptional profiles and functions are represented in the population of TAMs in primary brain tumors (43, 87).

TAMs are generally described with the permissive language of “M2-like”, sometimes specifically associated with the M2d subset. The M2d phenotype is associated with angiogenesis, matrix remodeling and immunosuppression (88). However, TAMs can simultaneously express gene signatures from multiple subsets including canonical M1 signatures, and some TAM subsets are more closely aligned solely with M1 phenotypes (89–91). Additionally, TAM maturation has been shown as distinct from typical alternative activation of macrophages (92). Although initially aligned most strongly with tumor-associated inflammation, it is now known that TAMs interact with cancer cells, other immune cells, and stromal cells to facilitate several other hallmarks of cancer (10, 93). TAMs help induce or access vasculature, promote invasion and metastasis, help avoid immune destruction and contribute to sustained proliferative signaling (93, 94).

TAMs have complex interactions in the TME with cancer cells. TAMs and cancer cells engage in various constitutive feedback loops, in which cancer cells recruit TAMs *via* factors such as colony stimulating factor-1 (CSF-1, a major driver of macrophage maturation). In turn, TAMs then secrete cellular factors such as protein kinase C, which upregulates production of CSF-1 from colon cancer cells (95). Similar feedback loops have been established in gastric cancer (hypoxia induced C-X-C motif chemokine ligand [CXCL]8 production from TAMs stimulates IL-10 secretion from cancer cells resulting in M2 polarization), breast cancer (reciprocal secretion of GM-CSF and C-C motif chemokine ligand [CCL]18 recruits TAMs and induces epithelial-mesenchymal transition), and others (96–98). Many of these same pathways reveal how TAMs promote the progression and metastasis of cancer cells. Once recruited, TAMs produce factors that remodel the TME to better support cancer cells, increase invasion *via* epithelial-mesenchymal transition, improve tumor vascularization, increase intravasation and extravasation, improve survival of circulating tumor cells, and help prepare the pre-metastatic niche. For example, TAMs produce matrix metalloproteinases and cathepsins, which disrupt cell junctions and basement membranes to aid in tumor invasion into surrounding tissues and blood vessels (99, 100). Another interesting example demonstrates that primary tumor secretion of factors such as vascular endothelial growth factor-A and transforming growth factor-beta induce distant lung endothelium to produce a variety of monocyte chemoattractants, thus preparing the metastatic niche (101). Each of these processes are complex, and detailed reviews have been written (96, 102, 103). The unique metabolism of TAMs also supports cancer cell growth. Their handling of glucose, amino acids, and lipids increases the availability of critical nutrients such as iron in the TME, contributes to immunosuppression, and provides strong growth signals to cancer cells (55, 104). Their universal metabolism of L-arginine, for example, results in extracellular depletion, rendering it unavailable to auxotrophic T cells that require it for proliferation (105).

The crosstalk between TAMs and cancer cells does not exist in a vacuum. Stromal cells and other immune cells are intimately involved in these processes as well. The biomechanical properties of the extracellular matrix (such as stiffness, topography, etc.) in the TME contributes to TAM polarization and function (106). Stromal cells themselves can recruit and polarize TAMs. Cancer-associated fibroblasts recruit TAMs, support M2 polarization, and inhibit cancer fighting immune cells from entering the TME (107, 108). Mesenchymal stromal cells, in addition to directly promoting tumor progression, secrete various angiogenic factors that polarize TAMs to an M2-like phenotype (109). TAMs are also involved in constant, reciprocal feedback with other immune cells. In hepatocellular carcinoma, for example, tumor-associated neutrophils recruit TAMs and regulatory T cells (Tregs) to the TME, promoting the immunosuppressive environment (110). In the TME, TAMs inhibit CD4+ and CD8+ T cells and natural killer (NK) cells, induce Treg differentiation, and recruit natural Tregs (111). Exhausted T cells and TAMs have been shown to maintain a positive feedback loop which supports the persistence of both populations in the TME (112).

There is some evidence from the veterinary literature supporting how macrophages may contribute to the immunosuppressive TME. Hartley et al. evaluated programmed death-ligand 1 (PD-L1) expression in primary canine MDMs, showing that expression was significantly induced by exposure to IFN-γ (113). Although IFN-γ is classically considered a pro-inflammatory cytokine, it is better understood now to have both immunostimulatory and immunosuppressive functions as well (114). Upregulation of PD-L1 on MDMs after exposure to IFN-γ may therefore be one mechanism of immunosuppression in the TME (113). Eto et al. demonstrated that damage-associated molecules released from necrotic canine cancer cells exerted immunosuppressive effects on macrophages *via* prostaglandin E2. This was hypothesized to be one mechanism by which dysregulation of the response to inflammation may contribute to tumorigenesis (115).

TAMs serve as prognostic biomarkers in many cases, as well as predictive biomarkers for response to treatment. TAMs are also associated with resistance to chemotherapy, immunotherapy, endocrine therapy, radiation therapy and targeted therapy (116–118). One meta-review of the prognostic significance of TAMs in human solid tumors identified a negative effect of TAM density on overall survival (OS) in gastric, breast, bladder, ovarian, oral, and thyroid cancer. In contrast, a positive effect was noted for OS in colorectal cancer, and no effect of TAM phenotype was found for any tumor type (119). However, the literature on this topic is vast, and as expected there are many conflicting conclusions. **Table 2** outlines key prognostic indicators of TAMs in select human and canine cancers. It can be difficult to comprehensively apply conclusions regarding TAMs and prognosis to large groups of patients, as the immune composition of the TME varies widely not only between tumor types, but also within tumor type dependent on patient factors such as age, sex, and body weight (146–148).

**Table 2:** Prognostic impact of tumor-associated macrophages in select human and canine cancers.

Cancer Type	Increased TAM infiltration associated with (human):	Increased TAM infiltration associated with (canine):
**Glioma**	Variable (120–122)	High-grade disease (123, 124)
**Osteosarcoma**	Increased OS and metastasis PFS in most studies; some exceptions noted (125–127)	Increased DFI (128)
**Lymphoma**	Advanced stage, decreased OS (129, 130)	High-grade disease (131)
**Soft tissue sarcoma**	Variable, but generally high-grade disease and decreased DFS and OS (132, 133)	High-grade disease and increased MI (134)
**Breast/mammary carcinoma**	Decreased OS and DFS, high-grade disease, HR-negative disease (135, 136)	Decreased OS, high metastatic rate, high-grade disease, HR-negative disease in most studies; one exception noted (137–141)
**Colorectal carcinoma**	Variable (associated with both improved and poor prognosis) (142, 143)	No association in one study, higher TAMs in normal tissue versus tumor in another (144, 145)

TAM, tumor-associated macrophage; OS, overall survival; PFS, progression free survival; DFI, disease free interval; DFS, disease free survival, MI, mitotic index; HR, hormone receptor.

Single-cell RNA-seq (scRNA-seq) techniques, first reported in 2009, have made incredible progress in elucidating the complexity of TAMs *a priori* (91, 149, 150). In brief, these techniques resolve the transcriptomes of single cells otherwise masked in bulk sequencing techniques. Several recent publications have explored the complexity and heterogeneity of TAM subsets across many cancer types using scRNA-seq (91, 151). A consistent finding across studies is that individual TAMs can express both canonical M1 and M2 gene profiles at the single-cell level (43, 91). A review article that attempted to integrate the findings of multiple scRNA-seq studies on TAMs proposed names for seven TAM subsets with distinct transcriptional profiles and metabolic pathways across many cancer types. For example, angio-TAMs had signatures enriched for angiogenesis, while IFN-TAMs were enriched for interferon-regulated genes and M1 markers. These seven subsets were not exhaustive, with many other subsets found in fewer numbers of cancers (152).

scRNA-seq of TAMs has not only been used to characterize new subsets of macrophages but also to identify macrophage-based prognostic markers or personalized treatment strategies (153, 154). For example, malignant gliomas in people are characterized by robust angiogenesis, a process aided significantly by TAMs. It has been demonstrated that the contribution of MDMs and TRMs to this process differs between primary and recurrent tumors, and therefore strategies targeting blood-derived TAMs versus resident microglia may have varying degrees of success at different time points in the course of disease (43, 155). Single-cell sequencing has also progressed beyond RNA, with new multi-omics techniques providing information at the single-cell level on epigenetic modifications, including histone modifications, DNA methylation, and chromatin openness (156). For example, combining epigenetic information with scRNA-seq data has produced data on TAM chromatin accessibility and *cis*-regulatory sequences (152). Single-cell sequencing data in companion animal species is in its infancy, although there are increasing numbers of publications in the past few years (157–162). ScRNA-seq data on canine TAMs has not yet been published to the authors’ knowledge.

## TAMs in companion dogs

In companion dogs, a large body of work exists regarding TAMs and mammary carcinoma due to its relative frequency and relevance as a translational model for breast cancer. Several groups have investigated the molecular crosstalk and changes in gene expression that occurs between canine mammary carcinoma cells co-cultured with macrophages (163–165). One notable conclusion was that carcinoma cells co-cultured with canine macrophages had increased migration and invasion compared to carcinoma cells in monoculture (163). Another group showed that carcinoma cells inhibited LPS activation of co-cultured macrophages, suggesting cancer-mediated immune suppression (164). Another demonstrated that TAMs increased the secretion of pro-angiogenic factors from canine mammary cancer stem-like cells (165). Multiple groups have published on associations between higher levels of TAMs in canine mammary tumors and shorter OS, higher metastatic rate, higher histologic grade, higher levels of intra-tumoral vascular endothelial growth factor and hormone receptor negativity (137–140, 166). One study found that, in contrast, higher numbers of macrophages in canine mammary carcinoma samples correlated with longer survival time. However, when taking the M2 TAMs alone a correlation with lymph node metastasis was noted (141). It should be noted that M1 and M2/TAM markers vary by study, and so results should be interpreted considering this fact. Some of the discrepancies arise from differing use of the term TAM, to either signify all macrophages in the TME versus only those with a pro-tumorigenic/M2-like phenotype. **Table 3** summarizes markers investigated for canine macrophages in the veterinary literature (most of which have been chosen based on human and murine data and have not been validated as able to discern between macrophage phenotypes in the dog).

**Table 3:** Markers investigated in the veterinary literature for canine macrophages.

Antigen	Used to identify	Polarizing agents used (various combinations)	Method used if successful to discern between M1/M2	Method attempted if unsuccessful to discern between M1/M2	References
CD80	M1	GM-CSF, LPS, IFN-γ		FC	(73)
CD86				FC	(73, 74)
CD40				FC	(74)
CD16				IF	(72)
CD32				IF	(72)
iNOS			IF	IF, FC	(72, 74, 131, 137)
MHC II*				IF, FC	(72, 74)
SOCS-3					(141)
LXN				IF	(72)
CD204	M2	M-CSF, IL-4, IL-13			(128, 131, 138, 140, 167–170)
CD206			IF	FC	(72–74, 137)
FcϵRI			FC		(73)
TGM2			IF	FC	(74)
SOCS-1			IF		(74, 141)
CD301					(164)
CD163				IF	(72, 123, 131, 168)
Arg-1				IF	(72, 74)
MS4A2				IF	(72)
Calprotectin/MAC387	M1, TAMs, recently recruited macrophages				(123, 131, 137, 139, 144, 166, 168)
IBA-1	Pan-myeloid, TAMs				(124, 134, 138, 167, 168, 170–172)
CD11/CD18	Pan-leukocyte, TAMs				(145, 173, 174)

*Has also been used as a general marker of macrophage activation (164, 175). CD, cluster of differentiation; iNOS, inducible nitric oxide synthase; MHC II, major histocompatibility complex class II; SOCS, suppressor of cytokine signaling; LXN, latexin; FcϵRI, high-affinity IgE receptor; TGM2 transglutaminase 2; Arg-1, arginase-1; MS4A2, membrane spanning 4-domains A2 (beta subunit of IgE receptor); MAC387, clone name commonly used that recognizes calprotectin; IBA-1, ionized calcium-binding adapter molecule 1; TAMs, tumor-associated macrophages; GM-CSF, granulocyte-macrophage colony-stimulating factor; LPS, lipopolysaccharide; IFN-γ, interferon-gamma; M-CSF, macrophage colony-stimulating factor; IL, interleukin; IF, immunofluorescence; FC, flow cytometry.

TAMs and their significance have also been investigated in other canine tumor types. Correlations between increased TAM infiltration and higher-grade disease has been identified in canine gliomas, lymphoma, soft tissue sarcomas and mast cell tumors (123, 124, 131, 134, 171, 173). In an analysis of canine hemangiosarcoma tumors, 67% contained macrophages that co-expressed an M2 marker and PD-L1. These tumors with PD-L1-expressing M2 macrophages had lower numbers of T cells in the TME. Additionally, M2 polarization and PD-L1 expression could be induced by tumor-conditioned media (167). In samples of canine tumors, pulmonary metastases from hemangiosarcoma were shown to have greater numbers of monocytes compared to metastases from other tumor types, as well as significantly higher CCL2 production, a monocyte chemoattractant protein (also called MCP-1) (174). In canine oral melanomas, M2 macrophages were significantly higher in malignant disease and associated with nuclear atypia and mitotic count. M2-marker CD163 positivity by itself was associated with metastatic disease and tumor-related death (168). In contrast to the above studies, in canine osteosarcoma, a higher percentage of M2 macrophages was correlated with a longer disease-free interval in one study. This same group analyzed paired primary tumor and metastasis samples of canine osteosarcoma and found the metastases to have a significantly higher degree of T cells, B cells and M2 macrophages, suggesting a role of these cells in the metastatic immune environment (128, 169). A preprint study from the National Cancer Institute confirmed these findings, showing a potential benefit from abundant M2 macrophages in canine and human osteosarcoma by transcriptomic analysis. These authors suggest this may be due in part to the unique properties of bone, namely that cytokines responsible for M2 differentiation can also inhibit osteoclast formation (170). In canine colorectal cancer, one study found no significant difference in macrophage counts between control tissue, adenomas, and carcinomas, while another found lower levels of macrophages in adenomas and carcinomas as compared to controls (144, 145).

There is also a body of literature that investigates the prognostic value of peripheral blood monocytes in dogs with cancer. Peripheral blood may provide insight into the TME’s systemic immunosuppressive impacts and can provide useful prognostic information in many tumor types. On analysis of peripheral blood, MCP-1 was increased in dogs with histiocytic sarcoma and lymphoma compared to healthy controls, while dogs with osteosarcoma were shown to have decreased chemotactic function of peripheral blood monocytes compared to controls (176–178). Increased monocyte counts or decreased lymphocyte-to-monocyte ratios have also been shown to be poor prognostic factors in canine lymphoma and osteosarcoma, similar to findings in many human cancers (178–182). A retrospective study of adjuvant carboplatin in dogs with hemangiosarcoma found an increased median survival time (MST) in dogs whose monocyte counts decreased post-operatively compared to those whose counts increased (265 days versus 66 days, respectively) (183). Interestingly, recent attention has been paid to circulating macrophage-like cells in peripheral blood. One veterinary study found that of 39 complete blood counts from dogs with circulating macrophage-like cells, 46% had a diagnosis of cancer (including both histiocytic and non-histiocytic origin) (184). These cells have the potential to be detectable in higher numbers than circulating tumor cells as sources of phagocytized tumor DNA (185).

## Therapy (preclinical/human)

When targeting macrophages as an anti-cancer therapy, there are a few general approaches. Broadly, one may attempt to deplete the TME of TAMs, inhibit the recruitment of monocytes/macrophages to the TME, or re-educate TAMs from tumorigenic to anti-tumor. (Although these categories are discussed separately below, it is likely that many of these therapies impact different macrophage subsets in multiple ways). Since the early work of Evans, Alexander and Fidler (discussed above), significant progress has been made in macrophage-based therapies, with many ongoing clinical trials. **Table 4** outlines some noteworthy macrophage-based cancer therapies in people and dogs. As the understanding of myeloid cells in the TME progresses, several anti-cancer therapies initially understood to have other targets have subsequently been found to have significant impacts on TAMs, very likely contributing to the anti-cancer effect. Examples include paclitaxel (via TLR-4 activation similar to LPS), imatinib (via the M-CSF receptor), and ibrutinib (via Bruton’s tyrosine kinase inhibition) (200–202).

**Table 4:** Select comparative macrophage-based cancer therapies in humans and dogs.

Therapy	Human	Canine
**CSF-1/R blockade**	Pexidartinib FDA approved for tenosynovial giant cell tumor, multiple phase I/II clinical trials ongoing for other agents (186, 187)	Canine blocking antibody developed (188)
**CCL2-CCR2 blockade**	Multiple phase I/II clinical trials ongoing (186)	Losartan investigated clinically in canine osteosarcoma and glioma (189–191)
**L-MTP-PE**	Failed FDA approval in 2007; approved for use in other countries for adjuvant therapy of osteosarcoma (192)	Early trials in canine hemangiosarcoma, osteosarcoma, mammary carcinoma and stage I oral melanoma (193–196)
**CD40 agonism**	Multiple phase I/II clinical trials ongoing (186)	Intralesional therapy with CD40 and IL-2 investigated clinically in dogs with STS (197)
**CD47/SIRPα blockade**	Multiple phase I/II clinical trials ongoing (186)	Xenograft model of canine DLBCL sensitive to combination anti-CD20 and CD47 blockade (198)
**CAR-M therapy**	NCT04660929 (first-in-human CAR-M phase I trial) (199)	Not applicable

CSF-1/R, colony-stimulating factor 1/receptor; CCL2-CCR2, C-C motif chemokine ligand/receptor 2; L-MTP-PE, liposome-encapsulated muramyl tripeptide phosphatidylethanolamine; CD cluster of differentiation; SIRPα, signal regulatory protein alpha; CAR-M, chimeric antigen receptor-macrophage.

### TAM depletion

Macrophage depletion has long been performed *in vitro* with bisphosphonates (BPs), initially agents of interest for anti-resorptive properties for skeletal metastasis (203–205). BPs are phagocytized by macrophages resulting in apoptosis and have been demonstrated *in vivo* to deplete TAMs and inhibit tumor growth (206, 207). They are still actively studied clinically for their macrophage-specific anti-cancer effects (208). There are other ways to selectively induce apoptosis in TAMs. Trabectedin, a marine-based DNA-binding small molecule, and its analogue lurbinectedin are approved for use in soft tissue sarcoma (trabectedin) and ovarian cancer and non-small cell lung cancer (lurbinectedin). While they likely have multiple mechanisms of action, they have preferential cytotoxicity for monocytes and macrophages *via* tumor necrosis factor-related apoptosis ligand (TRAIL) mediated apoptosis (209–211).

Another approach to TAM depletion is accomplished *via* blocking ligand-receptor interactions with small molecules or monoclonal antibodies. One popular strategy is the targeting of the M-CSF receptor, also called colony-stimulating factor 1 receptor (CSF-1R), or one of its ligands, M-CSF/CSF-1 (212). This pathway is responsible for the survival and differentiation of macrophages. Selective inhibitor of CSF-1R pexidartinib has shown significant clinical benefit for a rare tumor that overexpresses CSF-1 (tenosynovial giant cell tumor) (213). In other trials of CSF-1/R inhibitors for advanced solid tumors, stable disease or partial responses are observed with combinatorial therapies, and there are many other CSF-1/R inhibitors under investigation (212, 214, 215).

There are other interesting, experimental approaches to TAM depletion. One group engineered a hybrid peptide, consisting of melittin (a polypeptide that binds preferentially to TAMs) and a pro-apoptotic peptide. Injection of this hybrid peptide into a mouse model of lung carcinoma resulted in selective apoptosis of M2-like macrophages while sparing other immune cells (216). Chimeric antigen receptor (CAR)-T cells have been engineered to deplete TAMs *via* folate receptor beta binding, resulting in improved antitumor immunity and survival in a mouse model (217). Bi- and tri-valent T-cell engagers have been made that recognize CD3 on T-cells and a specific M2 marker on TAMs such as CD206 or folate receptor beta, resulting in selective T-cell mediated TAM depletion (218).

### Blocking TAM recruitment

Blocking the recruitment of MDMs to the tumor site is another therapeutic strategy. The chemokine CCL2 is crucial for the recruitment of c-c chemokine receptor type 2 (CCR2)-expressing monocytes to the TME. Of several compounds targeting CCL2/CCR2 to enter phase I clinical trials, CCX872, a CCR2-specific antagonist, showed the best anti-cancer activity, although the benefit was still modest. When given in combination with standard-of-care chemotherapy to advanced pancreatic cancer patients, it modestly improved OS in comparison to a historical cohort of patients receiving chemotherapy alone (219). Monoclonal antibodies against CCL2 have not been as effective (220). Another important pathway for monocyte recruitment is CCL5/CCR5. Several CCR5 antagonists in phase I clinical trials are repurposed drugs, initially developed for human immunodeficiency virus therapy (186, 221, 222). Therapeutic hurdles to blocking TAM recruitment include a rebound of recruited monocytes to the TME that can be seen after discontinuation of treatment, as well as redundancy in monocyte recruitment mechanisms (223, 224). However, decreasing recruitment or depleting TAMs can still enhance other immunotherapies, such as checkpoint inhibitors and cancer vaccines (225–227).

### TAM repolarization

TAMs may also be re-educated (or “repolarized”) from their generally immunosuppressive, pro-tumorigenic roles to have anti-cancer functions. Re-programming TAMs not only encourages macrophage-specific tumor cell killing but may also activate NK cells and T cells in the TME to kill tumor cells as well (228–231). In 2011, while testing CD40 activation (a co-stimulatory molecule expressed on antigen-presenting cells) on antitumor T cell responses, Beatty et al. found that CD40 agonism robustly activated macrophages in the TME, resulting in infiltration and depletion of tumor cells and tumor stroma (228). Since then, multiple agonists of CD40 have progressed to phase I/II clinical trials (186). A CD40 monoclonal antibody showed promising clinical in combination with chemotherapy for metastatic pancreatic adenocarcinoma (232). Checkpoint inhibitors are another avenue for TAM repolarization. TAMs, in addition to T cells, express PD-1, resulting in tumor immunity *via* PD-L1 expression from tumor cells (233). PD-L1 inhibition was shown to successfully rescue phagocytosis and re-educate TAMs in a mouse model (233, 234). Myeloid-specific checkpoint inhibitors have also been developed. Many tumor cells overexpress CD47, the prototypical “don’t eat me” signal that inhibits phagocytosis on normal cells from signal regulatory protein alpha (SIRPα)-expressing myeloid cells. This pathway can be blocked to increase phagocytosis of cancer cells and antigen presentation by macrophages, and promising clinical activity has been demonstrated in hematological malignancies and solid tumors (231, 235, 236). Increased Fc-mediated phagocytosis appears especially beneficial when combined with antibody-mediated opsonization (186, 237). As might be expected from a ubiquitously expressed self-marker, significant toxicity can be seen, and so different strategies have been employed to circumvent this limitation. Strategies include manipulation of the antibody structure or increasing specificity with a bispecific molecule target CD47 and a tumor antigen (186, 231). Other myeloid checkpoint inhibitor targets are under investigation such as the inhibitory leukocyte immunoglobulin-like receptor subfamily B member 1 receptor (LILRB1), an MHC class I-binding protein that suppresses phagocytosis, and CD24, another anti-phagocytic signal that binds through sialic-acid-binding Ig-like lectin (Siglec)-10 (238, 239). There are various other targets that repolarize TAMs including agonism or inhibition of TLRs, scavenger receptors (CD206, Clever-1, macrophage receptor with collagenous structure (MARCO), stimulator of interferon genes (STING), phosphoinositide 3-kinases gamma (PI3Kγ), or histone deacetylase (HDAC). Some of these therapies have broad impacts on anti-tumor immunity, although TAM re-polarization is recognized as an important contributing factor (186, 240, 241). A phase I/II clinical trial with a Clever-1 inhibiting antibody resulted in reversed immunosuppression in the TME and some clinical responses in patients (242). Conventional radiation therapy, proton irradiation, cryo-thermal therapy and cryosurgery have also been used to repolarize TAMs in addition to directly killing cancer cells. These therapies have multiple anti-cancer mechanisms, but in general cause activation of previously immunotolerant macrophages (243–246).

Toll-like receptors, a type of pattern recognition receptor, sense and respond to exogenous and endogenous danger signals. Their stimulation largely results in inflammatory, anti-cancer responses mediated by various immune cells including macrophages, and so make attractive targets for TAM repolarization. Historically, systemic administration of TLR agonists was inefficient and resulted in significant toxicity. However, with the advancements of nanomedicine, TLR agonists are able to be administered with fewer adverse effects, significantly improved tumor trafficking, and prolonged persistence in the TME (247, 248). Repolarization of TAMs has been investigated pre-clinically using nanoparticles (NPs) loaded or linked with various TLR agonists including CpG oligodeoxynucleotides, type I IFNs, imidazoquinolinone, and many others (247–252). Interestingly, empty NPs themselves have been shown to stimulate TLRs (247). As with many immunotherapies, combination therapy is of high interest. Imiquimod, a TLR-7 agonist, and anti-CD47 antibodies were delivered together on a nanoscale metal framework, leading to tumor eradication when combined with checkpoint inhibitors in a colorectal tumor model (253).

As mentioned above, the metabolism of TAMs and other myeloid cells in the TME is unique and contributes to immunosuppression and treatment resistance. Altering their metabolic pathways has therefore become an interesting therapeutic target. CB-1158, a small molecule developed to inhibit arginine metabolism and reverse immunosuppression in the TME, has entered phase I/II trials alone and in combination with checkpoint inhibitors (105, 254). Other interventions are under study that reprogram TAM metabolism towards an M1 phenotype, including suppressing glycolysis (resulting in decreased lactate in the TME), inhibiting hypoxia, and regulating iron handling. As might be expected, metabolic manipulation can have complex downstream effects, and the metabolism of TAMs even within a solitary tumor is significantly heterogenous (81, 104).

Cell-based therapies with macrophages deserve special mention. Combining cell therapy with nanotechnology has been used in of interesting ways to target TAMs. The first approach is to load M1-like macrophages with a variety of drug-laden NPs, inducing tumor cell killing *via* both the NP load and their natural M1 functions (255, 256). The second approach involves using NPs to deliver messages to TAMs to induce repolarization. One group used mannose-coated nanoparticles to introduce mRNA encoding M1-polarizing transcription factors into TAMs, subsequently inducing an M1 phenotype with anti-tumor properties (257). These approaches can be combined, for example, by using doxorubicin-laden NPs anchored to macrophages *via* LPS. When these reached their target, tumor cells were killed *via* M1 macrophages and doxorubicin, and TAMs were re-educated *via* the LPS (258). Macrophages have been modified in a variety of other ways as well for cell-based therapy, including engineered IFN-γ-laden “backpacks” and lentivirus-driven genetic engineering to express therapeutic IL-12, a pro-inflammatory cytokine (259, 260). Most interestingly, macrophages have been identified as candidates for CAR therapy. Large numbers of macrophages can be obtained after monocyte-apheresis or from HSCs (261, 262). Despite initial technological challenges, macrophages can now be engineered to express CARs against a cancer-specific antigen, called CAR-M cells (263, 264). It has been suggested that CAR-M cells possess an advantage over CAR-T cells in their ability to infiltrate into the TME and bypass the immunosuppressive environment, as well as re-educate “bystander” M2 macrophages (263, 265). Recently, the first-in-human CAR-M trial was launched, targeting human epidermal growth factor receptor 2 (HER2)-positive solid tumors (clinical trial NCT04660929). Several other targets are under preclinical investigation for CAR-M therapy (186, 264).

### TAM-specific imaging

Dynamic TAM-specific imaging is an important arm in treatment strategies. This has been investigated with both magnetic resonance imaging (MRI) and positron emission tomography (PET). TAM-targeting contrast agents used with MRI are phagocytosed by monocytes and then accumulate in tumors or leak directly into tumors due to the increased permeability of tumor vessels and are subsequently phagocytosed by macrophages. Alternatively, some agents are labeled with antibodies that bind macrophage receptors. Gadolinium, iron oxide nanoparticles and fluorine-19 have all been studied preclinically. Generally, these agents are unable to distinguish M1-like versus M2-like TAMs (266). Therapeutic uses of contrast agents have also been studied. One group used magnetic resonance targeting to direct iron oxide-labeled macrophages carrying an oncolytic virus to the sites of primary and metastatic tumors in mice. This resulted in increased macrophage infiltration and decreased tumor burden (267). With PET, radiotracers can be used to distinguish generally pro-inflammatory and anti-inflammatory macrophages. Tracers are in development for several prototypical M2 markers. Another approach is to target radiotracers to macrophage function, such as phagocytosis or antigen presentation (268). Imaging probes can be modified to include immunomodulatory therapeutics, resulting in imaging techniques that are both diagnostic and therapeutic. As discussed above, CSF-1/R blockade is a popular investigational approach for TAM depletion. Ligands of CSF-1R have been radiolabeled for PET tracers and investigated in mouse models (269). Several other targets of macrophage-based therapy are under similar investigation as potential imaging tracers. In theory, these tracers could be used to stratify patients pre-treatment who may benefit from macrophage-based therapy, deliver macrophage-targeted therapies and monitor response to therapy (269).

## Therapy (companion dogs)

In companion dogs, there are historically very few macrophage-specific therapies in cancer, although this is starting to change. As mentioned above, several therapeutics in use likely significantly impact TAMs, whether intentionally or not. As in humans, depletion of TAMs *via* BPs has been attempted. Liposomal clodronate (LC) was evaluated for the treatment of 13 dogs with soft tissue sarcoma (STS), and serial biopsies from 5 of them demonstrated significantly decreased numbers of infiltrating macrophages after the administration of LC. However, a decrease in TAMs was not correlated with tumor regression in this small sample (270). This group also used canine histiocytic sarcoma (HS) cells to evaluate sensitivity to LC *in vitro*, demonstrating apoptotic cell death in HS cell lines, but not other cell lines. They also showed tumor regression in 2 of 5 dogs with spontaneous HS treated with LC. As histiocytic neoplasms arise from DC or macrophage origin, the mechanism leading to apoptosis is likely similar to primary macrophages (271). They also demonstrated an increased sensitivity to chemotherapy in canine HS cells co-treated with clodronate (272). Regarding the CSF-1/R axis for TAM depletion, a blocking antibody against canine CSF-1R has been developed, although follow-up work has not yet been published (188).

Attempts to block the recruitment of TAMs have also been evaluated in dogs. Losartan, a type I angiotensin II receptor blocker, has recently gained interest as a newly recognized specific inhibitor of the CCL2-CCR2 axis mentioned above. Its ability to block monocyte/macrophage recruitment was initially evaluated in inflammatory and atherosclerotic diseases (273). Regan et al. were the first to evaluate its anti-cancer role as a TAM-targeting therapy in dogs. They initially demonstrated a reduction in pulmonary metastasis in a mouse model associated with a significant decrease in monocytes recruited to the lung through the inhibition of CCL2 (189). A follow-up study demonstrated that losartan inhibited monocyte migration to human and canine osteosarcoma cells *in vitro*. A prospective clinical trial evaluating safety and efficacy in combination with toceranib was performed on dogs with metastatic osteosarcoma. In the high-dose cohort, 4 of 16 dogs experienced a partial response of their lung metastasis for a median duration of 163 days, and another 4 dogs experienced stable disease for a median of 139 days (190). These findings were significant as toceranib alone had been shown to have minimal activity in metastatic osteosarcoma in dogs (274). This same group used losartan in combination with propranolol (shown to have MDSC depletion activity) and a cancer stem cell vaccine in canine glioma. Of 10 dogs (6 with high-grade tumors and 4 with low-grade tumors), 2 experienced a partial response and 8 had stable disease, with an overall MST of 351 days (191). This MST is comparable to other studies for high-grade disease (275, 276).

The final approach of re-polarizing or activating TAMs has also been investigated (largely unintentionally). In all examples noted below, other than when explicitly stated, macrophage-specific data were not reported, even when other immune cells were profiled. Several bacterial-based immunotherapies which cause broad immunomodulation in the TME undoubtedly impact TAMs in some way. Starting in the 1890s, a New York City surgeon Dr. William Coley was treating cancer patients with systemic or intra-tumoral injections of bacteria and bacterial products referred to as Coley’s toxins. Although he went through several iterations, the most successful concoction was heat-killed streptococcal organisms and *Serratia marcescens*, importantly anaerobic organisms that would flourish in a hypoxic tumor (277). Although his results eventually came under fire for inconsistencies, the kernel of his ideas has lived on in other experiments (278, 279). Importantly for macrophages, Coley’s toxins and other bacterial products are known to activate TLRs and other pathogen-associated molecular pattern receptors (280, 281). This bacterial activation of macrophages is the precursor to more specific TLR agonists used in macrophage-based therapies presently (240, 280).

Multiple bacterial-based anti-cancer therapies have been evaluated in dogs. These include a phase I trial of *Salmonella typhimurium* in dogs with spontaneous tumors, intra-tumoral injection of *Clostridium novyi* spores in dogs with spontaneous tumors (largely STSs), and *Listeria*- and *Salmonella*-based vaccine strategies in dogs with osteosarcoma (278, 279, 282–284). Two vaccine-based treatments for canine OS showed promise (one with immunogenic peptides from *Salmonella*-infected canine osteosarcoma cells and one with a HER2-targeting *Listeria*). Improvements in time to metastasis and survival were seen compared to historical cohorts receiving the standard of care in these preliminary studies (282, 284). Bacillus Calmette-Guerin (BCG), a live-attenuated strain of *Mycobacterium bovis*, is thought to activate macrophages predominantly through TLR-2, although it introduces a massive cytokine response and likely works through other mechanisms as well (285, 286). In the 1970s and 1980s it was used in a wide variety of canine cancers with varying degrees of success, although the treatment of transmissible venereal tumors seemed most promising (287–289). A more recent study used it in combination with vincristine in dogs with transmissible venereal tumors, resulting in shorter times to regression and increased macrophages in post-treatment biopsy samples (290).

Like the theory that the intentional administration of bacterial products may be beneficial in cancer, the survival benefits of post-limb salvage surgical site infections in dogs and people with osteosarcoma are well-established. This is likely attributable in part to the upregulation of NK cells, monocytes, and macrophages (291–293). Laboratory studies have demonstrated that chronic bacterial osteomyelitis-mediated suppression of tumor growth can be abrogated by the depletion of NK cells and monocytes (294).

Another example is liposome-encapsulated muramyl tripeptide phosphatidylethanolamine (L-MTP-PE), a synthetic derivative of a bacterial cell wall component. The base compounds, muramyl dipeptide or tripeptide, were studied in the 1980s by Fidler’s group, who demonstrated that these compounds activate monocytes and macrophages to kill tumor cells (295, 296). L-MTP-PE is a ligand of the Nod-like receptor and is taken up *via* phagocytosis, increasing inflammatory cytokines such as tumor necrosis factor alpha (TNF-α), IL-6, and C-reactive protein (193, 297). It appeared to improve OS and disease-free interval compared to the standard of care in dogs with osteosarcoma, hemangiosarcoma, and stage I oral melanoma in several studies from the 1990s (193, 194, 297). A similar study in dogs with mammary carcinoma did not show any benefit to L-MTP-PE treatment but did report the mild toxicity observed (similar in people), namely fever and shivering for 10-24 hours after dosing (195). Interestingly, this early work in companion dogs assisted the advancement of this compound through phase II/III clinical trials in people, and while it is approved for use in dozens of countries outside the USA, it was denied approval by the FDA in 2007 (192).

Other ways to re-polarize TAMs have also been investigated in dogs. A canine monoclonal agonist antibody against CD40, the macrophage-activating receptor, has been developed. In a phase I dose escalation trial, 27 dogs with STS were treated with intralesional agonist anti-CD40 canine antibody and IL-2. A clinical benefit was observed in 13 of 19 evaluable dogs at one month, including 2 partial responses. Seven of the 11 dogs with stable disease at one month continued to have stable disease for at least 50 days (197). As mentioned above, paclitaxel, imatinib, and BTK inhibitors can repolarize TAMs, and have been used in companion dogs with cancer (298–301). Chloroquine, an anti-malarial drug, also repolarizes TAMs *via* calcium-mediated nuclear factor kappa B (NF-kB) activation and was evaluated in combination with doxorubicin for dogs with lymphoma (302, 303). Of a total of 30 dogs, the response rate for this combination therapy was 93.3% and the median progression-free interval (PFI) was 5 months (302). Although head-to-head trials are needed, a retrospective evaluation of doxorubicin alone showed a similar PFI and a response rate of 84% (304). Another group treated canine MDMs with all-*trans* retinoic acid (ATRA) to reduce immunosuppressive secretions from the macrophages, with possible applications to reversing immunosuppression in the TME (305). There is human data, however, that shows ATRA may support M2 polarization (306).

Cancer vaccines rely in part on the presentation of tumor antigens to innate immune cells, including macrophages (307). In addition to the bacterial-based vaccines mentioned above, several other cancer vaccines have been looked at in veterinary medicine. An oncolytic vaccinia virus was used in xenograft models of canine STS and prostatic cancer, significantly increasing tumor macrophage infiltration and causing tumor regression (308). Similarly, an oncolytic herpes virus was used for canine gliomas. Pre-treatment biopsy samples showed significant infiltration with macrophages, and transcriptome analysis indicated myeloid cell activation after treatment (172). A whole-cell autologous cancer vaccine for metastatic canine hemangiosarcoma utilized a protein immune adjuvant known to activate macrophages and other immune cells (309, 310). Dogs with hemangiosarcoma received surgery followed by either chemotherapy or the vaccine; MSTs were the same (142 days) and either therapy conferred a benefit over surgery alone (309). Monophosphoryl lipid A, a TLR-4 agonist, has been evaluated as an adjuvant for anti-cancer vaccines in people, and an *in vitro* study showed it can activate canine macrophages as well (175, 311). Other examples abound (312–316).

Cytokine-based therapies have obvious applications to macrophage strategies as they are major drivers of macrophage polarization and function. Kurzman et al. exposed canine pulmonary alveolar macrophages to recombinant canine TNF-α and IFN-γ and found increased cytotoxicity against osteosarcoma cells (317). A phase I trial evaluating PEGylated TNF-α in dogs with spontaneous tumors showed increased tumor blood flow after administration and was well-tolerated. Minor or transient responses in melanoma, squamous cell carcinoma and mammary carcinoma were noted (318). Inhaled IL-2 and IL-15 have also been used to treat dogs with pulmonary metastatic disease, and a statistically insignificant increase in macrophage numbers in bronchoalveolar lavage samples was noted after IL-2 therapy (319, 320). These latter cytokines are largely studied for their anti-tumor activation of T cells and NK cells, although there is evidence that they activate macrophages as well (321–323).

Checkpoint inhibitors are areas of ongoing research interest in veterinary medicine. Although PD-1/PD-L1 blockade is most associated with T cells, as mentioned above TAMs may also express PD-1 and/or PD-L1. The blockade of this checkpoint has been shown to increase tumor cell phagocytosis by PD-1-positive TAMs (113, 324). Canine monoclonal antibodies blocking this axis have been developed and used in a pilot clinical study (325–329). Similarly, the CD47/SIRPα axis is conserved in dogs. A xenograft model of canine diffuse large B-cell lymphoma was sensitive to combinatorial anti-CD20 and CD47 blockade therapy (198).

## Conclusions and future directions

Tumor-associated macrophages have undeniable impacts on cancer progression and response to therapy. Veterinary therapeutics targeting macrophages and other myeloid cells lag behind human therapies, although progress is being made in recent years. As with other cell-based therapies, the cost of macrophage adoptive cell therapy will likely be a major obstacle in veterinary species. Regardless, there is a strong potential to use companion dogs as a streamlined pipeline for macrophage-based therapy discovery due to similarities in spontaneous cancers and immune systems between both species. When other immune parameters are being studied, monocyte/macrophage-specific data should also be collected whenever possible. In polarization experiments, precise and complete reporting of experimental conditions should be provided to improve reproducibility. Many topics tangentially related to TAMs are routinely studied in veterinary medicine, including radiotherapy, nanomedicine, and advanced imaging such as PET. Future studies should explore these topics in relation to TAMs in companion species, including the impact of radiotherapy on TAM polarization, the use of NPs to reverse immunosuppression in the TME and improve outcomes in veterinary oncology patients, and the use of novel PET tracers to explore macrophage-based imaging. Additionally, scRNA-seq techniques should be employed to deepen the understanding of TAMs in dogs and other companion species.

## Author contributions

RB drafted this review article, revised it critically, provided approval for publication, and agrees to be responsible for all aspects of this work. DT conceptualized this review article, revised it critically, provided approval for publication, and agrees to be responsible for all aspects of this work. All authors contributed to the article and approved the submitted version.
